# Intrahepatic biliary cystadenoma: Confusion, experience, and lessons learned from our center

**DOI:** 10.3389/fonc.2022.1003885

**Published:** 2022-11-10

**Authors:** Yongguang Yang, Weihuang Chen, Haiqiang Cen, Zuobiao Li, Xiaoqing Di, Yongjun Wu, Lijuan Liu

**Affiliations:** ^1^ Department of Hepatobiliary Surgery, The Affiliated Hospital of Guangdong Medical University, Zhanjiang, China; ^2^ Department of Pathology, The Affiliated Hospital of Guangdong Medical University, Zhanjiang, China; ^3^ Department of Radiology, The Affiliated Hospital of Guangdong Medical University, Zhanjiang, China; ^4^ Department of Ultrasound Diagnostics, The Affiliated Hospital of Guangdong Medical University, Zhanjiang, China

**Keywords:** intrahepatic biliary cystadenoma, hepatic cysts, intraoperative frozen section, hepatectomy, malignant transformation, recurrence

## Abstract

**Background:**

Intrahepatic biliary cystadenoma (IBC) is a rare benign cystic tumor of the liver. So far, it has not been comprehensively understood, which causes incorrect diagnosis, treatment confusion, and even inappropriate treatment. Here, we reviewed clinical data of IBC patients in our center, shared our experiences and lessons learned, and improved the level of diagnosis and treatment.

**Methods:**

The clinical data of 10 patients with pathologically diagnosed IBC, admitted to the Department of Hepatobiliary Surgery of the Affiliated Hospital of Guangdong Medical University from January, 2007, to January, 2022 were retrospectively analyzed.

**Results:**

10 patients underwent surgery and were discharged successfully. Cyst morphology: multiple cysts: 6 cases (6/10), monocular cyst: four cases(4/10). Six patients (6/10) were diagnosed as IBC preoperatively and received hepatectomy. Four patients with monocular cyst IBC underwent intraoperative frozen section examination, except one case showed IBC; the rest were misdiagnosed as simple liver cyst. In three misdiagnosed patients, one underwent open left hepatectomy seven days after the initial operation. The other patient refused to undergo reoperation and required follow-up observation. The last patient could not tolerate hepatectomy due to insufficient residual liver volume and chose follow-up observation

**Conclusion:**

For IBC, especially monocular IBC, it is easy to be misdiagnosed as simple hepatic cyst, which brings great confusion to clinical treatment. We propose strengthening communication with pathologists to deepen understanding of IBC. Attention should be paid to the cyst wall’s shape and the cyst fluid’s properties during the operation to avoid the missed diagnosis, misdiagnosis, or even improper operation. For suspicious cases, directly choose hepatectomy to avoid reoperation after thoroughly evaluating the patient’s condition.

## Introduction

IBC is a rare benign cystic tumor of the liver with carcinogenic potential. The tumor originates from the bile duct epithelium and is lined by mucus-secreting columnar or cuboidal epithelium. Due to the low incidence of atypical IBC, lack of specific clinical symptoms and signs, and insufficient attention for inexperienced surgeons, IBC is easily confused with other cystic liver diseases; therefore, treatment is often delayed or inappropriate. The clinical data of ten patients with pathologically confirmed IBC admitted to the Department of Hepatobiliary Surgery of the Affiliated Hospital of Guangdong Medical University between January, 2007. January 2022 was reviewed and reported to share our single-center experiences and lessons learned to help clinicians, especially young physicians, deepen their understanding of this rare disease and improve the level of diagnosis and treatment of IBC.

## Materials and methods

The clinical data of ten patients with pathologically confirmed IBC admitted to the Department of Hepatobiliary Surgery of the Affiliated Hospital of Guangdong Medical University between January, 2007 and January, 2022. This study was reviewed and approved by the Ethics Committee of the Affiliated Hospital of Guangdong Medical University.

## Results

### Clinical characteristics

Ten patients include four females and six males; male: female incidence ratio=3:2; age range 41–75 years; median age 61.2 ± 12.5 years. Clinical features: no discomfort (2/10), abdominal pain (6/10), skin and sclera jaundice (2/10). One patient with hepatitis B virus surface antigen-positivity, and the other nine patients were negative for hepatitis virus markers. The detecting key levels of APF and CA125 were within the normal range; CA199 was abnormal in 4 patients (4/10), and CEA was also abnormal in 2 patients (2/10). The general information of patients is shown (**Table 1**).

**Table 1:** Clinical data of ten patients with IBC.

Case no.	Age/sex	Clinical findings	Tumorlocation	Tumorsize	tumor markers		Method of diagnostic Imaging	Preoperative diagnosis
					CA199(0-27.0U/ml)	CEA(0-5.0ng/ml)		
1	67 M	Abdominal mass (physical examination)7 days	Left lateral lobe	10cm×7cm ×7.4cm	3.3	4.5	MRI/CDUS	IBC
2	50M	Abdominal mass (physical examination) 3 days	Right lobe	48mm×34mm ×49mm	15.0	7.3	CT/MRI/CEUS	IBC
3	41F	Abdominal mass (physical examination) 2 years, right upper abdominal pain 1 month	Right lobe	15cm×10cm ×14cm	5.8	3.6	CT/CEUS	Hepatic cystic tumor: liver cyst?
4	66 M	Right upper abdominal pain 2 months	Left medial lobe and right Anterior lobe	17cm× 14cm ×15cm	10.5	14.3	CT/CDUS	Hepatic cystic tumor: liver cyst?
5	62 M	Abdominal mass(physical examination) 7 years, right upper abdominal pain 2 months	Right lobe	8.5cm×8.3cm ×7.3cm	32.3	2.5	CT/MRI/CEUS	IBC
6	42 F	Abdominal mass(physical examination) 4 years, right upper abdominal pain 3 months	Left lateral lobe	8.2cm×8.3cm ×10.3cm	33.9	1.8	CT/CEUS	Hepatic cystic tumor: liver cyst?
7	75 F	Right upper abdominal pain 10 years,	Right lobe	8.2cm×8.9cm ×7.3cm	2.20	1.6	CT/CDUS	IBC
8	65 M	Skin and sclera jaundice 10 days	Left medial lobe and right anterior lobe	13cm×6.5cm ×6cm	44.5	4. 5	MRI/CDUS	IBC
9	74 M	Skin and sclera jaundice 10 days	Right anterior lobe	8cm×10cm ×6cm	6.2	2.7	CT/MRI/CDUS	Hepatic cystic tumor: liver cyst?
10	70 F	Abdominal pain 6 months	Left lateral lobe	9cm×7cm ×7.4cm	39.3	3.5	CT/MRI/CDUS	IBC

MRI, Magnetic resonance imaging; CT, Computed Tomography; CDUS, Color Doppler ultrasound; CEUS, contrast-enhanced ultrasonography; CA 19-9, carbohydrate antigen 19-9; CEA, carcinoembryonic antigen; IBC, intrahepatic biliary cystadenoma.

### Preoperative imaging of patients

**Figure 1:**
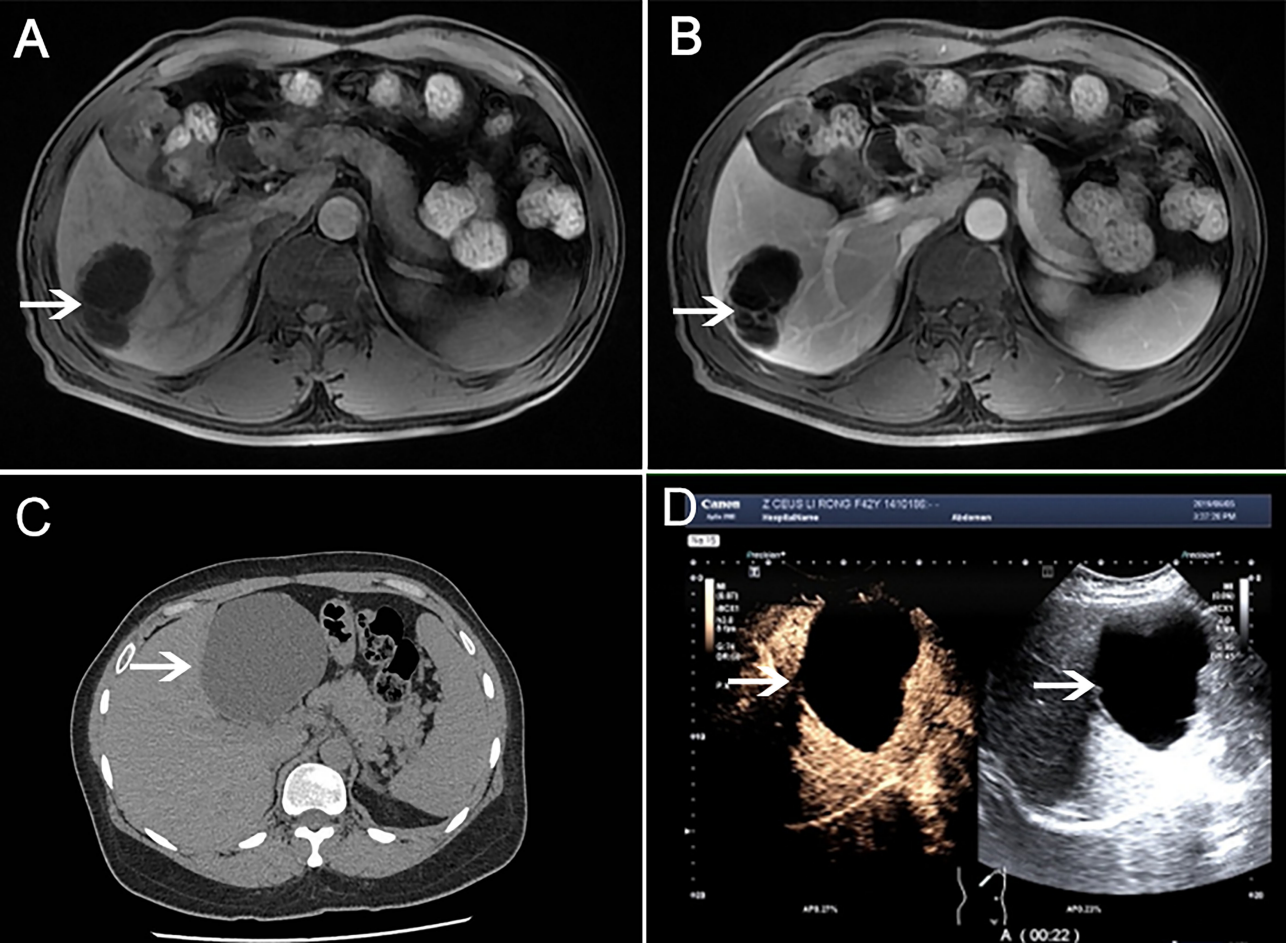
MRI examination (Case 2). **(A)** Multilocular cystic mass in the liver’s right lobe (shown by white arrow). **(B)** Contrast-enhanced T1-weighted images showed that the cyst wall was separated and enhanced, and there was no nodule on the cyst wall, and the contents of the lesion were not enhanced in portal vein phase. CT examination (Case 6). **(C)** A round low-density shadow is seen in the left lobe of the liver, with clear edges and uniform density in the capsule. **(D)** Contrast-enhanced liver ultrasound showed cystic space occupying the left outer lobe of the liver, with clear borders, good sound penetration in the cyst, and floating sheet echoes in the local area. There was no enhancement in the cysts at various stages after injection of contrast agent.

Patients were further imaged by abdominal imaging. Color Doppler ultrasound(CDUS) or contrast-enhanced ultrasonography(CEUS) was performed in all ten patients (10/10). Magnetic resonance imaging (MRI) examination was performed in six cases (6/10). Eight patients(8/10) underwent preoperative computed tomography (CT) examination. Tumor location: right lobe (5/10) (**Figures 1A, B** Case 2), left lobe (3/10) **(** **Figures 1C, D** Case 6), and left medial and right anterior lobe (2/10). Tumor morphology: multilocular cysts (6/10), monolocular cyst (4/10). The tumor size ranged from 3.4 to 17.0 cm in diameter, with a median diameter of 10.5 ± 6.7 cm. The patients’ general information and imaging data are detailed in **Table 1**.

### Surgical management and complications

All patients underwent surgical treatment. Six cases of multilocular cyst were preoperatively diagnosed with IBC, five cases directly underwent hepatectomy, and right anterior lobectomy + cholecystectomy + cholangiojejunostomy Roux-en-Y anastomosis in one case. Four patients with monocular cyst IBC underwent intraoperative frozen section examination, only one case was diagnosed as IBC, and the rest were misdiagnosed as a simple hepatic cyst. One patient with frozen section showing IBC had undergone laparoscopic fenestration and drainage of liver cyst + cyst wall biopsy, right hemihepatectomy (open conversion); Three patients with misdiagnosed as simple liver cyst, one patient had undergone single-port laparoscopic fenestration and drainage of liver cyst, and open left hemihepatectomy after seven days; the other patient underwent laparoscopic fenestration and drainage of liver cyst + cyst wall biopsy. Fenestration and drainage of liver cyst + cyst wall biopsy +common bile duct incision and exploration and T-tube drainage were performed on the last patient. Biliary fistula occurred in one case, which was cured by conservative drainage. A secondary operation cured one patient with abdominal gastrointestinal anastomosis caused by biliary-enteric anastomosis. All patients recovered and were discharged. The details are shown in **Table 2**.

**Table 2:** Operation method, intraoperative condition and follow-up results of ten cases of IBC.

Case no.	Operation method	Cystic wall morphology and cyst fluid properties	complication	Follow-up results
1	Resection of left lateral lobe of the liver	Multicystic mass with wall thickness of 0.1cm, jelly like cystic fluid in the cyst, rough wall, and grayish yellow lipid like deposit in the cyst	No	Alive/120months
2	Segment VIII+ partial segment V liver resection	Multilocular cystic mass with smooth wall and thickening of 0.1-0.2cm, with jelly like cystic fluid in the cyst	No	Alive/13months
3	Laparoscopic fenestration and drainage of liver cyst + cyst wall biopsy	Monolocular cystic mass, smooth wall, thickening 0.2-0.5cm, brown cystic fluid in the cyst, slightly turbid	No	Died/7 months
4	Laparoscopic fenestration and drainage of liver cyst + cyst wall biopsy, right hemihepatectomy (open conversion)	Monolocular cystic mass, wall thickening, smooth wall, with pale yellow cystic fluid in the cyst	Biliary fistula	Alive/33months
5	Right partial hepatectomy	Multilocular cystic mass with smooth wall and 0.2-0.8cm thickening, with jelly-like cyst fluid in the cyst	No	Alive/22months
6	Single-port laparoscopic fenestration and drainage of hepatic cyst, cyst wall biopsy, open left hemihepatectomy(7 days after the first operation)	Monolocular cystic mass with slightly thickened cyst wall, brown turbid flocculent liquid in the wall, and yellow fatty deposits in the wall	No	Alive/33months
7	Right partial hepatectomy	Multilocular cystic mass with smooth wall and thickening of 0.2-0.3cm, with gelatinous substance in the cyst	No	Alive/75months
8	Right anterior lobe resection +cholecystectomy +bile duct jejunal Roux-en-Y anastomosis	Multilocular cystic mass with smooth wall and thickening 0.1-0.2cm, with gelatinous substance in the cyst	Bleeding	Alive/90months
9	Fenestration and drainage of liver cyst + cyst wall biopsy +common bile duct incision and exploration and T-tube drainage	Monolocular cystic mass with smooth wall and thickening of 0.2-0.3cm, with clear liquid in the cyst	No	Died/27 months
10	Resection of left lateral lobe of the liver	Multilocular cystic mass with smooth wall and thickening of 0.1-0.2cm, with gelatinous substance in the cyst	No	Alive/113months

IBC, intrahepatic biliary cystadenoma.

### Intraoperative findings and postoperative pathology

**Figure 2:**
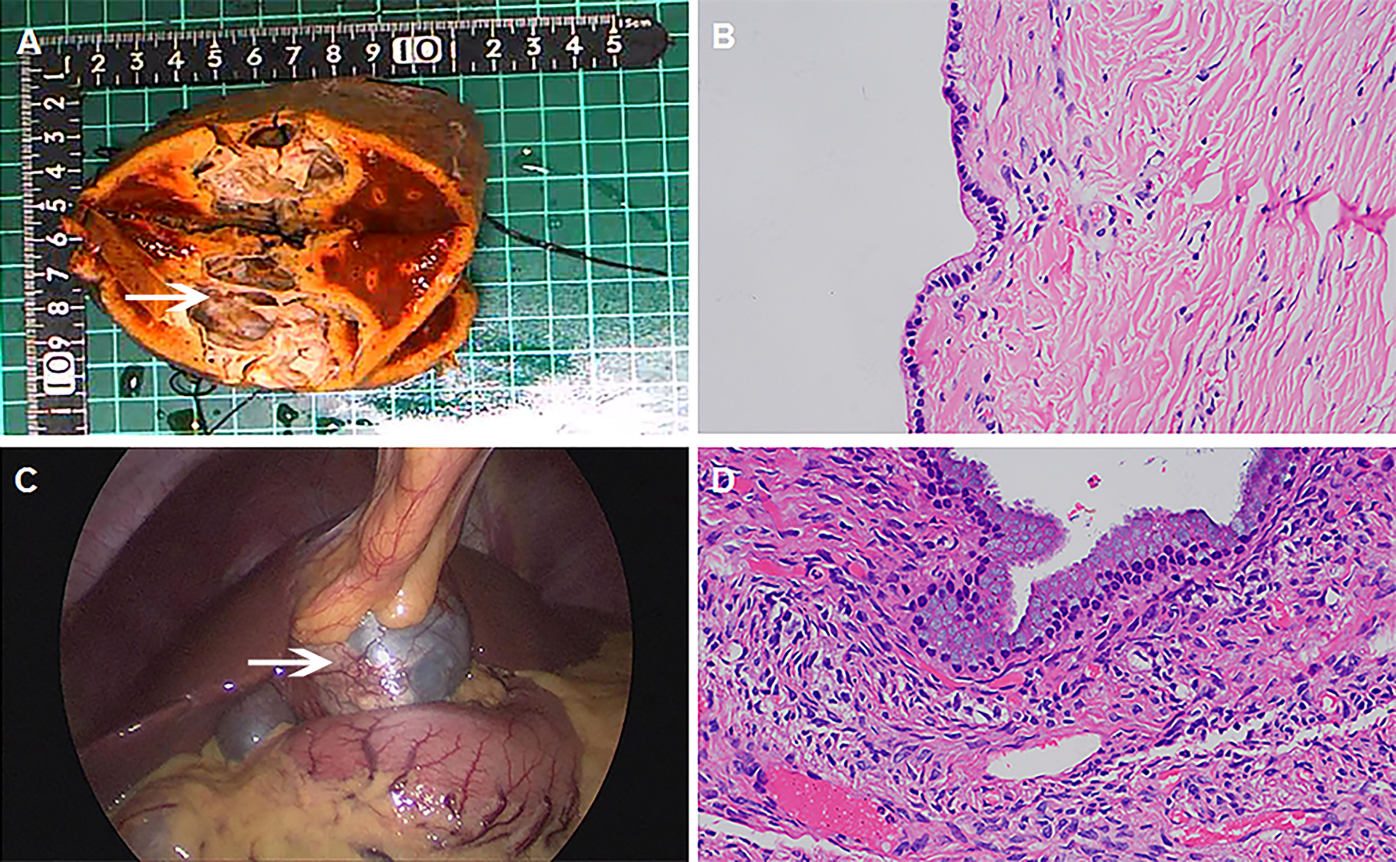
Intraoperative findings, postoperative specimens and pathology. (**A** Case 2) The resected specimen showed multilocular cystic structure (white arrow), with a wall thickness of 0.1-0.2cm and smooth inner wall. (**B** Case 2) Under the microscope, the inner wall of the cyst was covered with a single layer of cuboidal/columnar epithelium, with eosinophilic cytoplasm and nucleus at the base (HE 400 ×). (**C** Case 6) Laparoscopic exploration revealed yellow fat-like deposits in the cyst wall (white arrow), and turbid brown cyst fluid. (**D** Case6) Under the microscope, the cyst wall was composed of fibrous tissue, covered with monolayer or columnar, cuboidal epithelium, and ovarian-like stroma was seen locally(HE 400 ×).

The tumor was seen intraoperatively as a cystic lesion in all ten patients. There were four cases of monolocular cyst and six multilocular cysts. Cystic fluid properties: mucinous cystic fluid was observed in five cases (5/10), brown turbid cystic fluid in four cases (4/10), and the light yellow clear cystic fluid in one case(1/10). The thickness of the capsule wall ranged from 0.1 cm to 0.8cm. It was observed that the inner wall of the cyst was covered with a single cubic/columnar epithelium in five cases; the cytoplasm was lightly eosinophilic, and the nucleus was located at the base (**Figures 2A, B** Case 2). In three cases, yellow fat-like deposits were seen in the wall (**Figure 2C** Case 6). Pathology showed that the cyst wall was composed of fibrous tissue, covered with a single layer or columnar cubic epithelium, and ovarian-like interstitium was seen locally (**Figure 2D** Case 6). The details are shown in **Table 2**.

### Follow-up data

All patients in this group were followed up after surgery. The follow-up time was 13-120months, and the median follow-up time was 29.7 ± 48.2 months. The first IBC patient with monolocular died seven months after surgery due to recurrent tumor and malignant and extensive intra-abdominal metastasis. The second patient diagnosed with recurrent and malignant transformation of IBC 2 years after surgery died. The third patient was diagnosed with recurrent and malignant transformation of IBC 5 years after surgery, and the remaining liver volume was insufficient for surgical resection; the patient agreed to take apatinib and achieved partial response and progression-free survival of 60 months. The last patient underwent re-resection due to tumor recurrence in December 2019, seven years after the initial surgery. The remaining patients were followed up to date with no recurrence or malignant transformation, and the patients had a good quality of life. The details are shown in **Table 2**.

## Discussion

Intrahepatic bile duct cystic tumors (IBCTs) are a relatively rare type of cystic tumor of the liver, the etiology of which is still unknown and reported to account for 5% of all intrahepatic cystic diseases; more than 85% of patients are female (1, 2). There are only four female patients among the 10 cases, which may be related to this group’s limited number of cases. They are increasingly reported with a better understanding of IBCTs and advances in imaging techniques; however, their actual incidence should be higher because they are commonly misdiagnosed as simple liver cysts or other cystic lesions (3). Most of the lesions are solitary, mostly in the intrahepatic bile ducts and rarely in the extrahepatic bile ducts or the gallbladder (4), and most of the cysts are not identical to the bile ducts. IBCTs can be divided into two histological types: IBC and intrahepatic biliary cystadenocarcinoma (IBCA) (5, 6). IBC is a precancerous lesion with a malignant rate of up to 30% (3). Based on the nature of the lining epithelial envelope, they can be classified as mucinous and plasmacythematous, and mixed cystadenomas. In 1985, Emer defined cystadenoma of the intrahepatic bile ducts as an intrahepatic multilocular lesion with ovarian-like mesenchymal lining (5). However, in clinical practice, some cystadenoma specimens were not found to have ovarian-like mesenchyme (7). Only two of our group’s four female IBC patients had pathological findings of ovarian-like mesenchyme.

IBC has no specific clinical symptoms and signs, usually asymptomatic when compressing the surrounding tissues or blocking the bile duct, abdominal discomfort or pain may appear, and jaundice may appear when compressing the bile duct (8, 9). In this group, two patients with IBC were treated for jaundice due to cyst compressing the bile duct. The patient may show acute abdomen if the tumor ruptures with abdominal or internal bleeding. In a few cases, the combination of bile duct stones, pancreatitis, liver abscess, etc., may show corresponding symptoms.

IBC has no specific serological diagnostic markers, and the clinical findings show that the levels of CA19-9, CEA and CA125 are helpful for diagnosis (10).The level of CA19-9 in 60% of IBC and IBCA patients will increase, and CA19-9 will return to the normal level after the complete excision of the cyst. Therefore, the level of CA19-9 is considered a diagnostic and prognostic indicator (11–13).In our group, CA125 and AFP levels were normal in ten patients, while the CA19-9 level was elevated in two cases and CEA in two other cases. In addition to the role of CA19-9 in identifying cystadenoma, CEA was also found to be valuable in diagnosing IBC in this group of cases. It is reported in the literature that the misdiagnosed patients with simple liver cysts recurred after fenestration and drainage, and the cystic fluid CA19-9 continued to increase; the final resection pathology confirmed the IBC report (14). Invasive cyst fluid puncture carries the risk of bleeding and tumor spread; Cyst fluid CA199 detection is controversial in clinical practice and is recommended to be selective (7, 15).

The diagnosis and differential diagnosis of IBC mainly depends heavily on abdominal imaging examination. Color Doppler ultrasound is more sensitive at detecting septa in cystic lesions (16); CT more accurately demonstrates size and anatomic extent of these lesions (17, 18). MRI has better diagnostic value, and typical IBC showed multilocular cysts with irregular wall thickness and heterogeneous cystic fluid signal (6, 19). IBC appears as a hypointense lesion on T1W1 and hyperintense cystic fluid on T2W1, whereas the signal intensity may vary depending on the properties of the cyst fluid, protein concentration, and intracapsular hemorrhage (20). Enhancement scans show frequent enhancement of the cyst wall and compartment, with diminished enhancement in the portal vein and equilibrium phase. A significant thickening of the partition, papillary protrusions or wall nodules, gross calcification, or intracystic hemorrhage in the sac indicates hepatobiliary cystadenocarcinoma. MRI has advantages over CT in judging the shape of the cyst contents; magnetic resonance cholangiopancreatography (MRCP) can show if the cyst is connected to the bile duct, which helps formulate a surgical plan (21, 22). In addition, PEC-CT helps exclude IBCA (23). The trans-invasive examination or percutaneous fine-needle aspiration cytology is not recommended. The fluid component characteristics of the lesion have limited diagnostic value in addition to the risk of the risk dissemination along the needle tract (7).

Among these ten patients, six patients with multiple cysts were diagnosed as IBC preoperatively and directly underwent hepatectomy; four patients with monocular cyst IBC underwent intraoperative frozen section examination. Only one case was diagnosed with IBC, and the remaining patients were misdiagnosed as simple liver cysts, and received inappropriate treatment, cause treatment confusion and challenge. Similar misdiagnosis cases were also reported in other monocular IBC (14, 24, 25).

**Figure 3:**
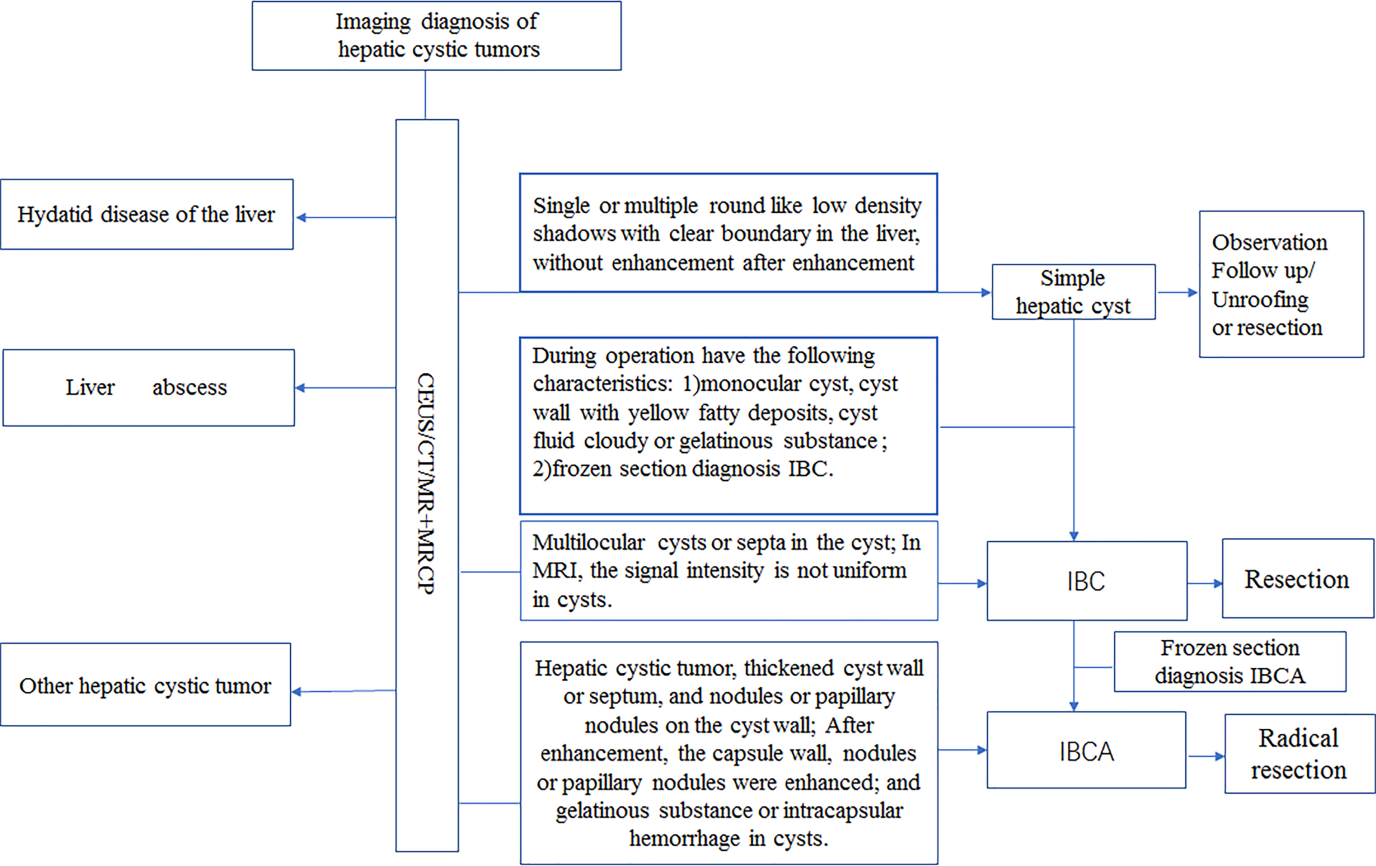
Classification and management of hepatic cystic tumor.

After analyzing and summarizing our center’s diagnosis and treatment, we share our single-center diagnosis and treatment experience: 1) For IBC, especially monocular IBC, it is easy to be confused with a simple liver cyst, which brings difficulties to clinical treatment. 2) In addition to imaging examination, intraoperative cyst wall morphology and cyst fluid properties are also significant for diagnosing IBC in operation. We believe that IBC is highly suspected of hepatic cystic tumor with the following characteristics (1): the cyst wall is thickened and uneven, or there are yellow fatty deposits on the cyst wall; (2) the cyst fluid is cloudy brown fluid, or the viscous and gelatinous substance in the cyst. 3) We recommend routine intraoperative frozen sections for all simple liver cysts. 4)For suspicious cases, under thoroughly evaluating the patient’s condition, directly choose hepatectomy to avoid reoperation. 5) Finally, we share the flow chart of the diagnosis and treatment of hepatic cystic tumor in our center(**Figure 3**).

In addition to informing patients of the limitations of the intraoperative frozen section, we also recommend strengthening the communication between the surgeon and the pathologist during the operation and timely feedback on the properties of cyst fluid and cyst wall morphology during the operation to improve the pathological accuracy of intraoperative frozen sections.

As early as 1892, Keen first reported the treatment of IBC by hepatectomy. So far, surgical resection is the first choice for treating IBC (15, 26). Due to the difficulty of the preoperative diagnosis of IBC and the high rate of malignant transformation, it is recommended that a possible diagnosis of IBC be considered in all patients with atypical hepatic cystic diseases. Since the disease is prone to recurrence, for any suspected IBC to be wholly removed, standard hepatic lobectomy should be performed as far as possible to ensure complete resection of the cyst wall and reduce the recurrence rate (27). Liver transplantation may be considered in cases where the disease involves the whole liver or incomplete enucleation (25). For IBC inappropriately treated with fenestration drainage, surgery should be performed as early as possible after functional evaluation. When IBC is suspected, we recommend multipoint pathologic biopsy, especially from any solid or nodular area. After adequate evaluation, direct hepatectomy rather than simple cyst drainage is recommended to avoid missed diagnosis and reoperation (14, 28). Owing to the recurrence of IBC, it is recommended that patients undergo examination regularly after the operation. Patients with recurrence can be operated actively, and advanced cases with recurrence and malignant transformation should be treated with radiotherapy, chemotherapy, Tyrosine kinase inhibitors, or hyperthermic perfusion, which is beneficial in alleviating terminal symptoms (29–31).

## Conclusions

IBC is a rare liver cystic tumor. It has no specific clinical symptoms or signs. Atypical IBC, especially monolocular IBC, has a high rate of missed diagnosis and misdiagnosis, requiring a fine clinical acumen. We recommend paying attention to cystic wall morphology and cyst fluid properties during the operation, strengthening communication with pathologists to deepen understanding of IBC, improving rapid pathological diagnosis, and avoiding the rate of misdiagnosis, misoperation, and reoperations.

## Data availability statement

The original contributions presented in the study are included in the article/supplementary material. Further inquiries can be directed to the corresponding author.

## Ethics statement

The studies involving human participants were reviewed and approved by the Ethics Committee of the Affiliated Hospital of Guangdong Medical University. The patients/participants provided their written informed consent to participate in this study.

## Author contributions

All authors read and approved the final manuscript. YY and ZL performed the surgery. WC wrote the original draft. HC, XD, and YW collected and arranged clinical, imaging, and pathological data. LL and YY designed the study and revised the manuscript. All authors contributed to the article and approved the submitted version.

## Funding

This study was supported by the Science Foundation of Guangdong Province (2018A030307076); Zhanjiang City Financial Fund Technology Special Competitive Allocation Project (2018A01037); National Natural Science Foundation of China “Breakthrough” Support Project (20301DFY20190157); Scientific research project of Guangdong Provincial Bureau of Traditional Chinese Medicine (20201189); Clinical Research Project of Affiliated Hospital of Guangdong Medical University (LCYJ2021B002).

## Acknowledgments

We are appreciative for support from the imaging department and pathology during the patient’s diagnosis, treatment, and our draft process. We are equally appreciative for the patient’s cooperation in our medical activity and the generous authorization of our report. Finally, we would like to thank Editage (www.editage.cn) for English language editing.

## Conflict of interest

The authors declare that the research was conducted in the absence of any commercial or financial relationships that could be construed as a potential conflict of interest.

## Publisher’s note

All claims expressed in this article are solely those of the authors and do not necessarily represent those of their affiliated organizations, or those of the publisher, the editors and the reviewers. Any product that may be evaluated in this article, or claim that may be made by its manufacturer, is not guaranteed or endorsed by the publisher.
